# Near complete genome sequences from Southern Vietnam revealed local features of genetic diversity and intergenerational changes in SARS- CoV-2 variants in 2020–2021

**DOI:** 10.1186/s12879-023-08814-8

**Published:** 2023-11-17

**Authors:** Anna S. Gladkikh, Thang M. Cao, Ekaterina O. Klyuchnikova, Manh H. Dao, Alena A. Sharova, Vasilina D. Melnichenko, Margarita R. Popova, Tatiana V. Arbuzova, Valeriya A. Sbarzaglia, Nadezhda A. Tsyganova, Edward Ramsay, Vladimir G. Dedkov

**Affiliations:** 1 Saint Petersburg Pasteur Institute, 14 Mira Street, Saint Petersburg, 197101 Russia; 2 Pasteur Institute in Ho Chi Minh City, Ho Chi Minh City, Vietnam; 3 Martsinovsky Institute of Medical Parasitology, Tropical and Vector Borne Diseases, Sechenov First Moscow State Medical University, Moscow, Russia

**Keywords:** COVID-19, SARS-CoV-2 lineages, Morbidity, NGS, Vietnam

## Abstract

**Background:**

Since its beginnings in 2019, the COVID-19 pandemic is still a problem of global medical concern. Southern Vietnam is one of the country's vast regions, including 20 provinces and the densely populated metropolis Ho Chi Minh City. A randomized retrospective study was performed to investigate the epidemiology and genetic diversity of COVID-19. Whole-genome sequencing of 126 SARS-CoV-2 samples collected from Southern Vietnam between January 2020 and December 2021 revealed the main circulating variants and their distribution.

**Methods:**

Epidemiological data were obtained from the Department of Preventive Medicine of the Vietnamese Ministry of Health. To identify circulating variants, RNA, extracted from 126 nasopharyngeal swabs of patients with suspected COVID-19 were sequenced on Illunina MiSeq to obtain near complete genomes SARS-CoV-2.

**Results:**

Due to the effectiveness of restrictive measures in Vietnam, it was possible to keep incidence at a low level. The partial relaxation of restrictive measures, and the spread of Delta lineages, contributed to the beginning of a logarithmic increase in incidence. Lineages 20A-H circulated in Southern Vietnam during 2020. Spread of the Delta lineage in Southern Vietnam began in March 2021, causing a logarithmic rise in the number of COVID-19 cases.

**Conclusions:**

Pandemic dynamics in Southern Vietnam feature specific variations in incidence, and these reflect the success of the restrictive measures put in place during the early stages of the pandemic.

**Supplementary Information:**

The online version contains supplementary material available at 10.1186/s12879-023-08814-8.

## Introduction

In late December 2019, a group of patients with pneumonia of unknown etiology was reported in Wuhan, Hubei province, China [1]. From there, it spread massively to all 34 provinces of China. The number of confirmed cases increased rapidly, and thousands of new cases were identified daily. The highest average newly-confirmed cases per day reached 3,000 [2]. Amidst this situation, the WHO declared the novel coronavirus outbreak a 'public health emergency of international concern' [3]. On February 11, the International Committee on Taxonomy of Viruses designated the novel coronavirus ‘SARS-CoV-2', and associated illness was designated 'COVID-19' [4]. In some cases, SARS-CoV-2 infection (COVID-19) can lead to a severe chronic respiratory syndrome with pneumonia and/or a myriad of other complications.

After applying many COVID-19 control measures (shutting down Wuhan on the 23rd of January, blocking all travel to and from the city), the daily number of cases in China subsequently decreased. In contrast, large clusters were reported in an increasing number other countries.

In Vietnam, the first case was identified on January 23, 2020 [5]. Since the beginning of 2020, Vietnam has experienced four waves of the COVID-19 epidemic: the first wave (January 23—July 24, 2020) with 415 cases, including 106 domestic cases and 309 imported cases; the second wave (July 25, 2020—January 27, 2021) with 1,136 cases, including 554 domestic and 582 imported; the third wave (January 28—April 26, 2021) with 1,301 cases, including 901 domestic and 391 imported; and a fourth wave (starting on April 27, 2021) which is still ongoing [6]. By May 4, 2023, the national COVID-19 caseload had reached 10,433,530 with 43,057 fatalities [7]. From March 2020 to December 2021, the strategy of COVID-19 control was tracing, quarantine, and PCR analysis of every single person who had been in contact with test-positive individuals. All people entering Vietnam were obligated to quarantine and underwent COVID-19 diagnostics (RT-PCR).

SARS-CoV-2 is an enveloped, positive-sense single-stranded RNA virus. Its 30 kilobase genome features a rapid mutation rate, and nucleotide changes accumulate over time. Identifying and following mutations helps to minimize adverse public health impacts. Southern Vietnam is one of three geographic regions which includes 20 provinces and a principal megalopolis, Ho Chi Minh, with a population of around 9 million people. Here, we studied the epidemiology of COVID-19, including whole-genome sequencing of SARS-CoV-2 samples from Southern Vietnam, during the period from January 2020 to December 2021. Identification of the main circulating variants during the pandemic in Vietnam and their succession is valuable information needed by scientists, policy makers, and health professionals.

## Materials and methods

### Epidemiological data and study materials

All epidemiological data were obtained periodically from the Department of Preventive Medicine, Vietnamese Ministry of Health [7, 8]. During the period from January 2020 to December 2021, nasopharyngeal swabs of patients with suspected COVID-19 from twenty southern provinces of Vietnam (Bac lieu, An Giang, Bạc Liêu, Bac ninh, Binh Duong, Cà Mau, Da nang, Đồng Nai, Hau Giang, Ho Chi Minh City, Hung yen, Kiên Giang, Kom tum, Lam Dong, Long An, Quang Ninh, Tây Ninh, Tien giang, Tây Ninh, Vinh long), and nine imported cases, were collected and delivered to the Pasteur Institute in Ho Chi Minh City. Swabs were collected in 500 µL of viral transport medium, transported in tripled sealed containers, and stored under ultralow temperature conditions until further analysis. Viral RNA was extracted, and samples were tested for the presence of SARS-CoV-2 by real-time polymerase chain reaction (PCR) using a standard procedure at the Pasteur Institute in Ho Chi Minh City.

### RNA purification, Real-time PCR

Total RNA from nasopharyngeal swabs samples were obtained by extraction and purification using the QIAamp Viral RNA Extraction Kit (QIAGEN, Germany) with the QIAcube Connect automatic station (QIAGEN, Germany) according to the manufacturers recommendations. Samples were eluted and stored at -70° C until further analysis. For SARS-CoV-2 detection and to assess viral load, swabs were thoroughly analyzed using LightMix® Modular Wuhan CoV E-gene reagents (Roche SAP) according to manufacturer’s recommendations. SARS-CoV-2-positive samples featuring Ct values of 25 or less were selected and studied further. 

### SARS-CoV-2 genome enrichment

To create NGS libraries, viral RNA was subjected to reverse transcription and subsequent PCR enrichment. Reverse transcription was performed using random hexamers with the Reverta L Kit (AmpliSens, Russia) following the manufacturer's protocol. Samples (cDNA) were stored at -20°C until amplification. To obtain a near complete genome sequences of SARS-CoV-2 (excluding the 5′ and 3′ ends), a total of 138 primer pairs covering the entire genome were applied according to a previously described multiplex PCR protocol [9]. Reactions were performed in a total volume of 25 μl containing 2 μl cDNA, 0.1 μM of each primer, and 12.5 μl 2 × BioMaster HS-Taq PCR mix (Biolabmix, Novosibirsk, Russia). PCR were performed with following parameters: 95 °C for 3 min; 40 cycles (93 °C for 10 s, 57 °C for 30 s, 72 °C for 30 s); a final extension (72 °C for 5 min). Reactions were performed in a C1000 Touch thermal cycler (Bio-Rad, USA). The products were analyzed by electrophoresis on a 2.0% agarose gel in the presence of ethidium bromide. Reaction products were purified using the AMPure XP purification kit (Beckman Coulter, UK) in 1:1 ratio according to the manufacturer's instructions and equimolary pooled. The concentration of the PCR-fragment mixture was measured using the dsDNA HS Assay Kit (Invitrogen, USA) with a Qubit 4.0 Fluorimeter (Invitrogen, USA) and used for library preparation.

### Library preparation and sequencing

Library preparation was performed using the TruSeq DNA CD Indexes Kit (Illumina Inc., USA) according to the Illumina TruSeq Nano DNA Kit protocol. Sequencing was performed on a MiSeq instrument using MiSeq V3 chemistry, generating 2 × 200 bp reads.

### Genome assembly

The quality of the Illumina reads was assessed using the FastQC program [10]. Sequencing, trimming and adapter removal were done using Trimmomatic-0.39 [11]. Trimmed reads were aligned to the SARS-CoV-2 Wuhan-1 reference genome (NC_045512.2) using the native Bowtie2 aligner [12]. Genomic variants were then called using the GATK pipeline [13]; variants were filtered by minimal quality (QUAL < 400). Consensus sequences for each sample were generated using bcftools consensus [14].

### Availability of data

Sequences were uploaded to GISAID under the following IDs for year 2020: EPI_ISL_812922, EPI_ISL_760247, EPI_ISL_17454567, EPI_ISL_17436319, EPI_ISL_17454569, EPI_ISL_654872-EPI_ISL_654874, EPI_ISL_17433648-EPI_ISL_17433661, EPI_ISL_654866-EPI_ISL_654870, EPI_ISL_654886-EPI_ISL_654889, and EPI_ISL_654875-EPI_ISL_654884. For 2021, the IDs were: EPI_ISL_16034578, EPI_ISL_16034555-EPI_ISL_16034573, EPI_ISL_16034575-EPI_ISL_16060862, and EPI_ISL_17433647.

###  Variant annotation and phylogenetic tree reconstruction

Variant calling files (.vcf) were processed for the effect of SNP variation with the SnpEff tool (http://pcingola.github.io/SnpEff/). Unique S gene genotypes were extracted from the set of annotated.gvcf files and used to make a variant map for each strain using the “Variant frequency plot” tool from Galaxy EU (www.usegalaxy.eu). A global phylogenetic tree of SARS-CoV-2 variants was constructed using the tools implemented in Nextclade [15].

## Results

### Epidemiology of SARS-CoV-2 in Southern Vietnam, 2020–2021

**Fig. 1:**
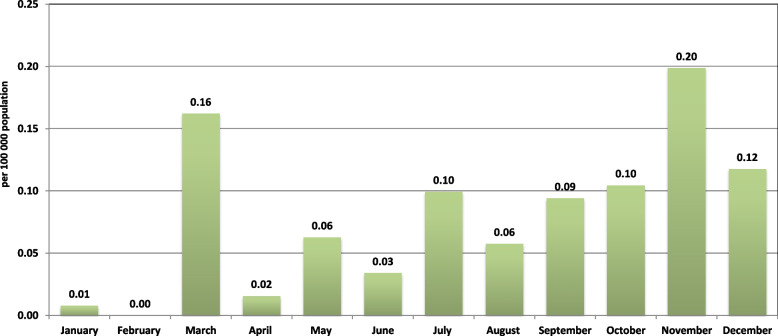
COVID-19 incidence rate in Southern Vietnamese regions in 2020. Due to restrictions on movement and border crossing under strict quarantine, the incidence in 2020 in Southern Vietnam was sporadic. The incidence did not exceed 0.2 per 100,000 population

The first COVID-19 cases were confirmed in Southern Vietnam on January 23, 2020, when two Chinese individuals arrived in Ho Chi Minh City and tested positive for the virus [16]. Viral dissemination in the community was prevented through effective public health strategies, which included mandatory quarantine for travelers from other countries, isolation of people with confirmed COVID-19, and isolation of contacts (those in contact with a confirmed COVID-19 case). The Vietnamese authorities took measures to close air borders and cancel all international flights in March 2020. Since then, only repatriates, foreign specialists, and highly skilled workers have been allowed entry into the country under the strictest quarantine conditions. In the context of the measures taken, incidence in Southern Vietnam in 2020 was sporadic. The incidence rate did not exceed 0.2 per 100,000 population (Fig. 1).

Considering the situation in twenty provinces of Southern Vietnam, a total of 365 cases of COVID-19 were registered in 2020. Five provinces did not detect a single case: An Giang, Binh Phuoc, Hau Giang, Lam Dong, and Soc Trang. Almost half of all cases occurred in Ho Chi Minh City, Southern Vietnam's largest city (Table 1).

**Table 1:** COVID-19 case numbers by province in 2020

No	Provinces	2020–01	2020–02	2020–03	2020–04	2020–05	2020–06	2020–07	2020–08	2020–09	2020–10	2020–11	2020–12	Total
1	An Giang	0	0	0	0	0	0	0	0	0	0	0	0	0
2	Bac Lieu	0	0	2	1	18	0	3	3	17	4	0	3	51
3	Ben Tre	0	0	1	0	0	0	0	0	0	0	0	0	1
4	Binh Dương	0	0	0	0	0	0	0	1	5	0	6	14	26
5	Binh Phuoc	0	0	0	0	0	0	0	0	0	0	0	0	0
6	Ba Ria- Vung Tau	0	0	0	0	0	9	23	7	8	12	9	0	68
7	Ca Mau	0	0	0	0	0	0	1	0	0	0	0	0	1
8	Can Tho	0	0	2	0	0	0	0	1	3	1	2	1	10
9	Đong Nai	0	0	0	1	0	0	0	2	0	1	0	3	7
10	Đong Thap	0	0	4	0	1	1	0	0	0	16	0	3	25
11	Hau Giang	0	0	0	0	0	0	0	0	0	0	0	0	0
12	Kien Giang	0	0	0	0	0	0	1	0	0	0	0	0	1
13	Lam Đong	0	0	0	0	0	0	0	0	0	0	0	0	0
14	Long An	0	0	0	0	0	0	0	0	0	0	0	1	1
15	Soc Trang	0	0	0	0	0	0	0	0	0	0	0	0	0
16	Tay Ninh	0	0	2	1	1	0	0	0	3	0	0	0	7
17	Tien Giang	0	0	0	0	0	0	0	0	0	1	1	0	2
18	Ho Chi Minh City	3	0	49	3	4	3	8	8	0	5	56	13	152
19	Tra Vinh	0	0	2	0	0	0	2	0	0	0	0	2	6
20	Vinh Long	0	0	0	0	0	0	0	0	0	0	2	5	7
Total		3	0	62	6	24	13	38	22	36	40	76	45	365

**Fig. 2:**
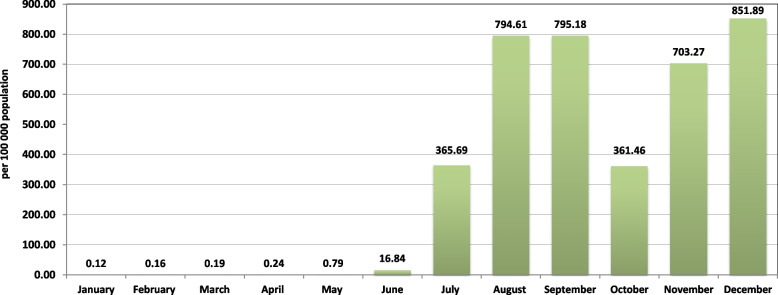
COVID-19 incidence rate in Southern Vietnamese regions in 2021. During the first five months of 2021, the incidence rate remained at a consistently low level. In May 2021, the incidence rate increased to 0.8 per 100,000 population. Since June 2021, there has been an increase in the number of cases from 16.8 in June to 851.9 by December 2021

In January 2021, there was a relaxation of the ban on air travel, but only under certain conditions: if there was evidence of having completed a full course of COVID-19 vaccination (no later than 14 days before the intended visit to the country) as well as a negative test performed not more than 72 h earlier. During the first five months of 2021, the incidence rate remained at a consistently low level. In May 2021, the incidence rate was already 0.8 per 100,000 population. Despite previously successful public health measures, there has been a dramatic increase in cases since June 2021. The incidence rate rose from 16.8 in June to 851.9 by December 2021 (Fig. 2). The largest increase in COVID-19 cases was observed in Ho Chi Minh City; the total number of reported cases reached 504,558 in 2021 (Table 2).

**Table 2:** COVID-19 case numbers by province in 2021

No	Provinces	2021–01	2021–02	2021–03	2021–04	2021–05	2021–06	2021–07	2021–08	2021–09	2021–10	2021–11	2021–12	Total
1	An Giang	0	0	0	3	0	27	300	1787	3172	6695	12,268	9903	34,155
2	Bac Lieu	1	0	0	0	1	0	35	115	266	3447	10,842	15,531	30,238
3	Ben Tre	1	0	12	6	0	1	832	937	124	562	5988	24,790	33,253
4	Binh Dương	5	3	7	2	3	494	20,356	103,283	93,980	42,667	19,293	7189	287,282
5	Binh Phuoc	0	0	0	0	0	2	217	324	839	439	6919	21,496	30,236
6	Ba Ria- Vung Tau	10	0	2	7	0	7	1575	2062	616	653	11,226	11,534	27,692
7	Ca Mau	0	0	4	3	0	0	32	130	227	1708	8457	30,192	40,753
8	Can Tho	0	1	0	0	0	2	1620	2580	1515	2278	18,424	16,011	42,431
9	Dong Nai	1	0	14	0	1	50	4031	21,559	23,126	17,493	20,829	9658	96,762
10	Dong Thap	0	2	0	1	1	134	3009	3954	1177	1602	14,304	20,822	45,006
11	Hau Giang	0	0	0	0	0	1	208	253	84	885	5054	7051	13,536
12	Kien Giang	0	7	12	18	0	2	256	1447	4345	5230	11,111	8630	31,058
13	Lam Dong	0	0	0	0	0	0	48	196	43	265	2876	6835	10,263
14	Long An	1	1	1	1	6	144	6121	18,104	7474	2490	3454	2125	39,922
15	Soc Trang	0	0	0	0	0	0	279	644	375	4785	13,375	11,146	30,604
16	Tay Ninh	4	3	9	11	1	9	1016	3568	2337	3749	38,910	33,871	83,488
17	Tien Giang	3	0	1	0	0	141	3187	8054	3388	2942	8217	8674	34,607
18	Ho Chi Minh city	17	43	10	41	287	5423	95,848	132,854	160,844	38,062	40,169	30,960	504,558
19	Tra Vinh	1	0	0	0	2	2	325	981	159	1423	6165	19,907	28,965
20	Vinh Long	0	0	0	0	0	3	613	1172	132	911	11,177	29,591	43,599
Total		44	60	72	93	302	6442	139,908	304,004	304,223	138,286	269,058	325,916	1,488,408

The maximum increase in the number of COVID-19 cases in the second half of 2021 was observed in the three provinces closest to Ho Chi Minh City. Binh Duong province recorded 287,282 total cases in 2021. The southern districts of Binh Duong province are very urbanized and are within one of the districts of Ho Chi Minh City. The population is about 2.5 million people. Currently, Binh Duong is a zone of ecotourism, alongside a focus on historical and cultural relics. In the provinces of Dong Nai and Tay Ninh, 96,762 and 83,488 cases were detected, respectively. Due to their large populations, proximity to Ho Chi Minh City, and natural mobility of the population, these provinces experienced a high rate of increase in COVID-19 incidence (Table 2).

### SARS-CoV-2 genetic diversity in Southern Vietnam, January 2020 to December 2021

**Fig. 3:**
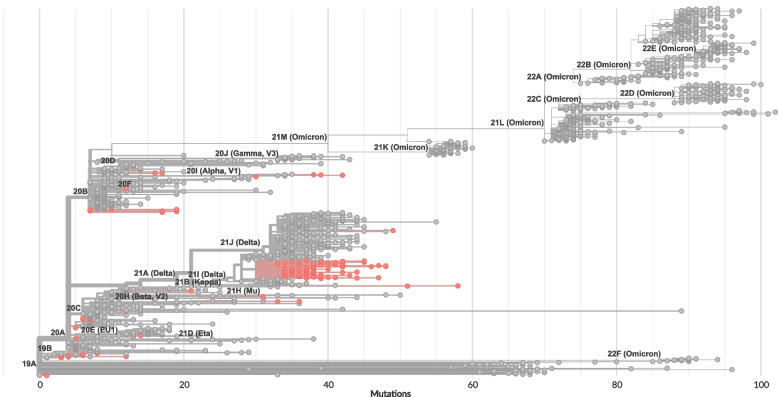
SARS-CoV-2 phylogenetic tree reconstruction based on Nextclade tools. Branches are labeled by lineage according to Nextstrain nomenclature (legend top left). Sequences obtained in this study are colored red. The scale bar at the bottom indicates the number of nucleotide differences between each sample and the Wuhan-Hu-1/2019 reference sequence (GenBank: MN908947)

Whole‐genome sequencing was performed for SARS‐CoV‐2 isolated from 9 imported cases and 117 domestic cases from 14 provinces in Southern Vietnam from January 2020 to December 2021. Several sequences from Northern Vietnam were analyzed as well. Genomic analysis results based on Nextclade SARS‐CoV‐2 Clade Assigner showed 11 clades including the Alpha, Beta, and Delta VOCs (Fig. 3, Table 3). The maximum number of mutations among non-structural genes was noted in ORF1ab. Among structural genes, the spike protein gene was foremost (Table 4). One of the first imported cases of SARS‐CoV‐2 (from China to Ho Chi Minh City) belonged to clade 19A.

**Table 3:** Geographic distribution of successfully sequenced samples

Year	Provinces	Nextstrain clades (VOC name, pango lineage)
2020	Bac Lieu	19A
	Binh Duong	19B
	Lam Dong	20A
	Tay Ninh	20B
	Ho Chi Minh city	20C
	Tien Giang	20D
	Tra Vinh	20E
	+ Northern Vietnam	20I (Alpha)
2020	1 imported case	19A
2021	An Giang Bac Lieu Binh Duong Ca Mau Dong Nai Hau Giang Kien Giang Long An Ho Chi Minh city Tien Giang Vinh Long	20A 21I (Delta, AY.57) 20H (Beta, V2)
2021	8 imported cases	20H (Beta, V2) 21A (Delta, AY. 30) 21J (Delta, AY. 85) 21I (Delta, AY. 57)

**Table 4:** Number of SNPs in each ORF in each lineage

Clade	ORF1ab	ORF3a	ORF6	ORF7a	ORF7b	ORF8	ORF10	S	E	M	N	NCR^a^
19A	12	1	0	0	0	0	0	1	1	0	3	5
19B	7	1	0	0	0	1	0	0	0	1	2	0
20A	13	4	1	0	0	0	0	5	0	1	3	1
20B	26	0	0	1	0	0	0	4	1	1	5	3
20C	10	2	0	0	0	1	0	1	0	1	0	1
20D	7	0	0	0	0	0	0	2	0	0	4	1
20E	5	1	0	0	1	0	1	3	0	1	1	1
20 H	14	3	2	0	0	2	0	6	1	1	6	4
20I	12	0	0	0	0	3	0	9	0	1	7	2
21A	18	1	0	3	0	1	0	10	0	2	6	5
21I	135	21	2	5	1	5	3	38	0	3	20	16
21 J	25	2	0	5	3	3	0	15	1	3	8	5

^a^non-coding region

### Mutations in the SARS-CoV-2 spike protein gene in samples from Vietnam

**Fig. 4:**
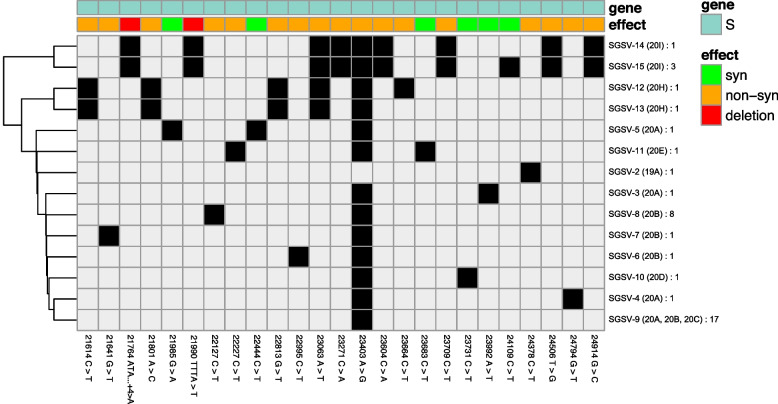
Phylogenetic comparison and heatmap analysis of variations observed in clades 19A-B and 20A-H. The figure shows the phylogenetic diversity and analysis of variations observed in lineages 19AB and 20A-H based on S gene sequence. Mutations were found relative to the Wuhan-Hu-1/2019 (GenBank: MN908947) reference strain. SGSV – S gene sequence variants. Clades in which this pattern occurs are indicated in parentheses. The number of strains of the specified variant in the studied group is indicated by a colon

**Fig. 5:**
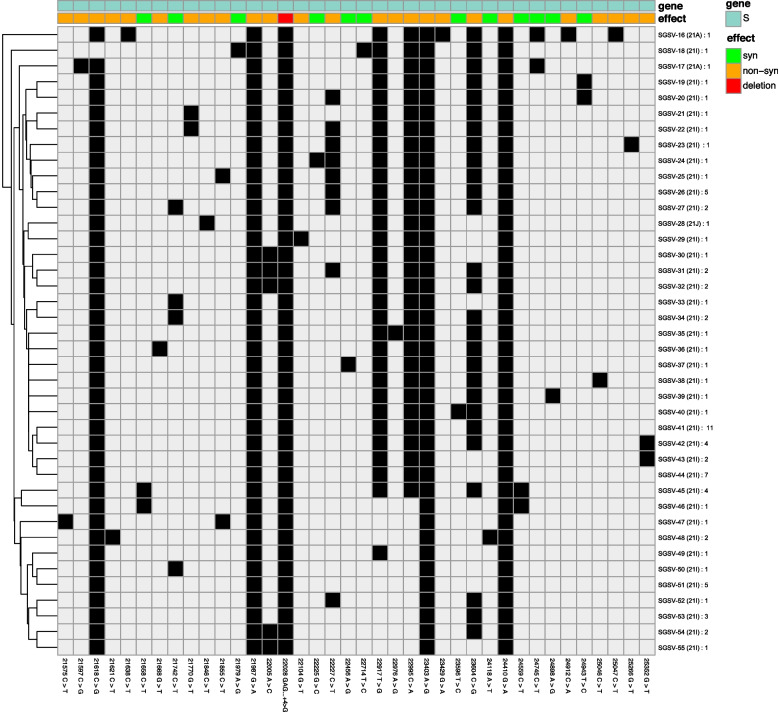
Phylogenetic comparison and heatmap analysis of variations observed in Delta clades. In 2021, most strains from domestic and imported cases were in the Delta VOC, major lineage 21I, and minor lineages 21A and 21J. The figure shows the phylogenetic diversity and analysis of variations based on S gene sequence. Mutations were found relative to the Wuhan-Hu-1/2019 (GenBank: MN908947) reference strain. SGSV – Sgene sequence variants. Clades in which this pattern occurs are indicated in parentheses. The number of strains of the specified variant in the studied group is indicated by a colon

Of 126 genomes that underwent sequencing, 55 S gene sequence variants (SGSV) were identified based on SNP pattern. SGSV-1 was identical to the Wuhan-Hu-1/2019 (MN908947) reference strain without any SNPs or indels. The remaining 54 SGSVs had at least one SNP compared to the reference (Figs. 4 and 5). Within clade 19B, no mutations in the S gene were identified; all studied samples belonged to SGSV-1. In clade 19A, one strain had a non-synonymous SNP in the spike gene (S939F) and was attributed to SGSV-2. The other four strains of the clade had S gene sequences identical to that of the reference (Wuhan-Hu-1/2019). In clade 20A, apart from the defining D614G substitution, there was a unique non-synonymous A1078S substitution. Based on S gene SNP patterns, strains of this clade belonged to four different SGSVs (Fig. 4). Clade 20B samples had only one defining mutation (D614G) and three unique non-synonymous variations: A27S, L189F, and T478I.

 Four different SGSVs were identified within the analyzed strains. Strains from clade 20C were identical by S gene sequence with only the single defining mutation (D614G), and they carried SGSV-9. Clade 20D samples had a single defining mutation (D614G) and a synonymous 23,731 C > T SNP. Clade 20E (EU1), apart from the defining A222V variant, had a synonymous 23,683 C > T (Fig. 4). Samples belonging to the Alpha VOC lineage (clade 20I), apart from defining mutations, gained a 24,109 C > T synonymous SNP. Beta VOC (20H) samples contained no defining D215G or E484K mutations, and no specific mutations.

In 2021, the majority of samples from domestic and imported cases belonged to the Delta VOC, major clade 21I, and minor clades (21A, 21 J). Altogether, forty SGSVs within the Delta clade were identified (Fig. 5). Apart from defining variations, Delta variant sequences contained some non-defining substitutions: L5F, V70F, T95I, and V1264L. According to Nextclade CoVariants, these mutations occur in Delta variants (21A, 21I, 21 J) with different frequencies: L5F (1.06—1.5%), V70F (0.97%), T95I (occurs in 21 J with frequency of 49.09%), and V1264L (1.5—15%). There are also unique non-synonymous substitutions: S12C, T20I, P26S, V36F, S98F, N148T, G181V, I472V, A623T, T1117K, P1162S, P1162L, and C1235F. Synonymous mutations include 21,658 C > T, 21,742 C > T, 21,979 A > G, 22,225 G > C, 22,456 A > G, 22,714 T > C, 23,596 T > C, 24,118 A > T, 24,745 C > T, 24,559 C > T, 24,943 T > C, and 24,898 A > G (Fig. 5).

## Discussion

COVID-19 continues to be a pressing public health problem. Different countries have had varying levels of success in combating the COVID-19 pandemic, with some countries among the most successful in the world at containing the pandemic and others in serious jeopardy. In the East Asia and Pacific regions, the most successful are considered Singapore, New Zealand, South Korea, China, and Vietnam [17]. Due to strict anti-COVID-19 measures and other actions of the authorities, the epidemic in Vietnam was somewhat under control, with a minimal number of registered cases spanning 1.5 years until July 2021. In early 2020, all passengers arriving on inbound flights to Vietnam underwent testing using real-time reverse transcription polymerase chain reaction (RT-PCR) (requirement expired 15 May 2022), alongside a mandatory 14-day quarantine in centralized facilities (requirement expired 1 January 2022) [18].

Genomic surveillance for SARS-CoV-2 was announced by the WHO as a necessary measure for pandemic control. In June 2020, the WHO Virus Evolution Working Group was established with a specific focus on SARS-CoV-2 variants, their phenotype, and their impact on countermeasures [19]. Genomic surveillance became the international standard for combating COVID-19 [20]. The scale and strategy of SARS-CoV-2 sequencing in different countries were implemented differently, which led to uneven data coverage across regions in the GISAID database and GenBank.

The Pasteur Institute in Ho Chi Minh City has shown vigilance and readiness in terms of monitoring SARS-CoV-2 lineages using NGS methods. According to the data obtained, it can be said that the first imported cases were from China and belonged to 19A (lineage B). Variants of lineage 19B (lineage A) circulated in Southern Vietnam from February to March 2020. In China, lineages A and B circulated together for a short time and were quickly totally replaced by lineage B [21]. Strains of lineage 19A (lineage B) were detected in Southern Vietnam until June 2020 and were further replaced by lineage 20 (A-E). Alpha and Beta (20I and 20H) variants from the VOCs list circulated in Southern Vietnam throughout 2020 without causing rises in the incidence or deterioration of the epidemiological situation. In neighboring ASEAN countries (Cambodia, Malaysia, the Philippines, Singapore, Indonesia, Thailand), lineage B variants widely replicated in 2020.

In addition to multiple introduced variants in 2020 [22], some countries have reported differences in the distribution and spread of local genetic variants. Indeed, local lineages have been reported in neighboring ASEAN countries [23–26].

The beginning of the pandemic was marked by two major lineages, denoted ‘A’ and ‘B’, which are probably the result of two different spillover events. The first zoonotic transmission likely involved lineage B viruses and occurred from late-November to early-December 2019 (no earlier than early November 2019). Introduction of lineage A likely occurred within weeks of the first event [27]. All published sequences from environmental samples taken at the Huanan Market also fall in lineage B. The earliest lineage A genomes had no direct epidemiological connection with the Huanan market [28].

At the beginning of 2021, the Alpha and Beta variants (20I, 20H) continued circulation in Southern Vietnam. Like global COVID-19 pandemic trends, they were completely replaced by Delta lineages, followed by a sudden increase in incidence marking the beginning of the 4th pandemic wave in Vietnam. The Delta variant was represented by three phylogenetic lineages (21A, 21I, 21J), with the 21I lineage dominating. Intragenomic variability within the Delta lineage was observed as a set of synonymous and non-synonymous substitutions in addition to the defining ones.

The first Delta variant sequences available in GISAID for ASEAN countries came from Malaysia [24] and Singapore in February 2021 [22]. From early 2021, Delta became the predominant variant in the Philippines, as well as in Singapore, Indonesia, Malaysia [25] and Thailand, causing a new wave of morbidity [29]. During 2021, the Delta variant, which included the original B.1.617.2 Pango lineage and descendants of the AY lineage, became widespread in Malaysia. Lineage B.1.617.2 was identified as an early dominant strain circulating throughout the country, but was later superseded by lines AY.59 and AY.79 [30]. In Southern Vietnam according to Pango classification, lineage AY.57 was dominating. The limitation of the study is that we observed and described only part of the genetic variation among SARS-CoV-2 lineages in Southern Vietnam in 2020–2021. The primary diversity, associated with multiple independent imports in 2020, was superseded by the Delta variant by mid-2021. Delta then became the main circulating variant in Vietnam, causing a new wave of incidence. Similar dynamics were observed in neighboring Asian countries. Watching subsequent waves of growth in case numbers associated with the easing of anti-COVID restrictions demonstrates the effectiveness and necessity of containment measures. Monitoring of circulating and emerging SARS-CoV-2 strains is essential to understanding the mechanisms of the pandemic and developing effective responses.

## Conclusion

Pandemic dynamics in Southern Vietnam feature specific variations in incidence, and these largely reflect the success of the substantial, ongoing restrictive measures put in place during the early stages of the pandemic. Tracking of circulating lineages revealed major variants from the list of variants-of-concern, including intragenomic variability within circulating lineages. Further evaluation of epidemiological features and the circulation of SARS-CoV-2 variants is an essential part of COVID-19 surveillance in Vietnam.

### Supplementary Information

**Additional file 1:  Supplementary Table.** Samples used in the study.

## Data Availability

Genomic consensus sequences obtained in this study are deposited in GISAID (https://www.gisaid.org/). The accession numbers for each sample submitted to and approved by CDC by IBX are publicly available in GISAID database. Raw data are available upon reasonable request from the corresponding author. The datasets used in the present study are available from the corresponding author on reasonable request.

## Abbreviations

SARS-CoV-2: Severe acute respiratory syndrome coronavirus 2
COVID-19: Coronavirus disease 2019
WHO: World Health Organization
NGS: Next generation sequencing
PCR: Polymerase chain reaction
RT-PCR: Real time polymerase chain reaction
RNA: Ribonucleic acid
VOC: Variants of concern
SNP: Single nucleotide polymorphism
ASEAN: Association of South East Asian Nations
