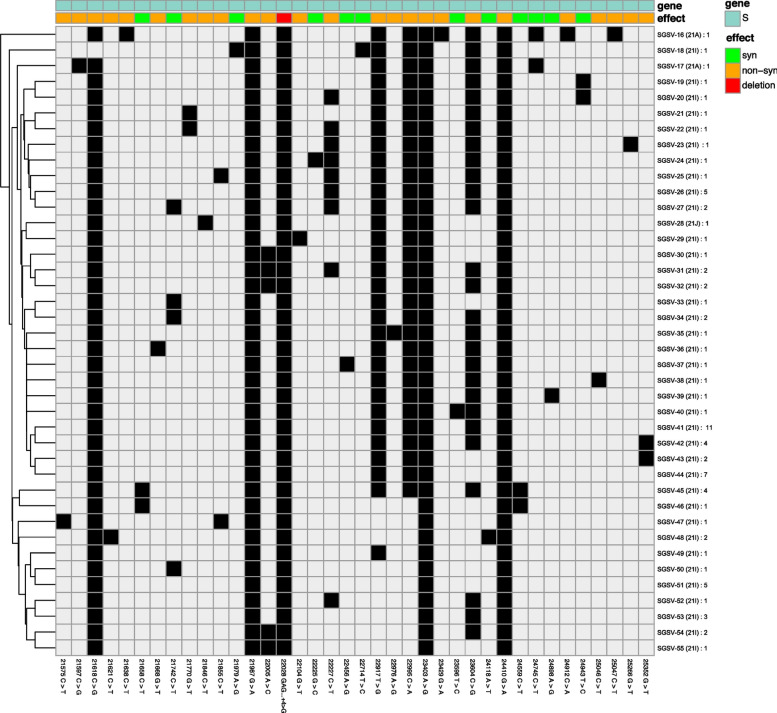# Correction: Near complete genome sequences from Southern Vietnam revealed local features of genetic diversity and intergenerational changes in SARS- CoV-2 variants in 2020–2021

**DOI:** 10.1186/s12879-023-08853-1

**Published:** 2023-12-21

**Authors:** Anna S. Gladkikh, Thang M. Cao, Ekaterina O. Klyuchnikova, Manh H. Dao, Alena A. Sharova, Vasilina D. Melnichenko, Margarita R. Popova, Tatiana V. Arbuzova, Valeriya A. Sbarzaglia, Nadezhda A. Tsyganova, Edward Ramsay, Vladimir G. Dedkov

**Affiliations:** 1 Saint Petersburg Pasteur Institute, 14 Mira Street, Saint, Petersburg, 197101 Russia; 2 Pasteur Institute in Ho Chi Minh City, Ho Chi Minh City, Vietnam; 3 Martsinovsky Institute of Medical Parasitology, Tropical and Vector Borne, Diseases, Sechenov First Moscow State Medical University, Moscow, Russia

**Correction: BMC Infect Dis 23, 806 (2023)**

**https://doi.org/10.1186/s12879-023-08814-8**

In the original publication of this article [1] Figs. 4 and 5 were swapped. The incorrect and correct information is shown in this correction article, the original article has been updated.

**Incorrect Figure 4**


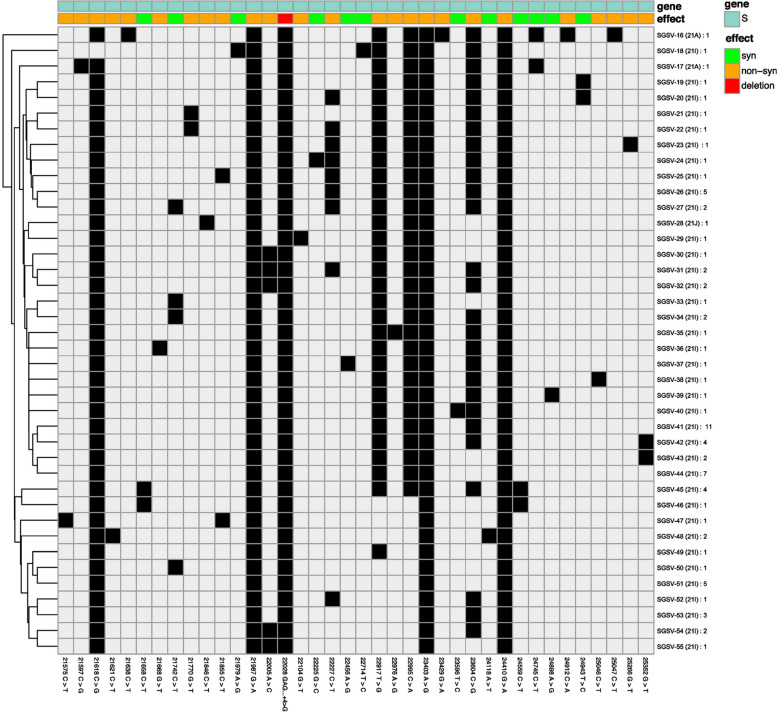


**Incorrect Figure 5**


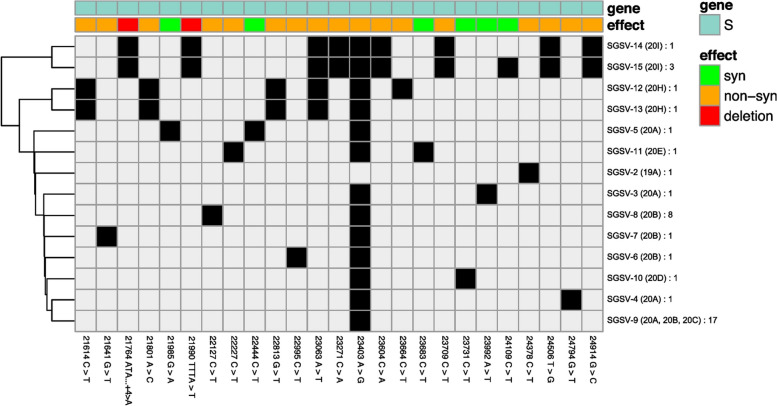


**Correct Figure 4**


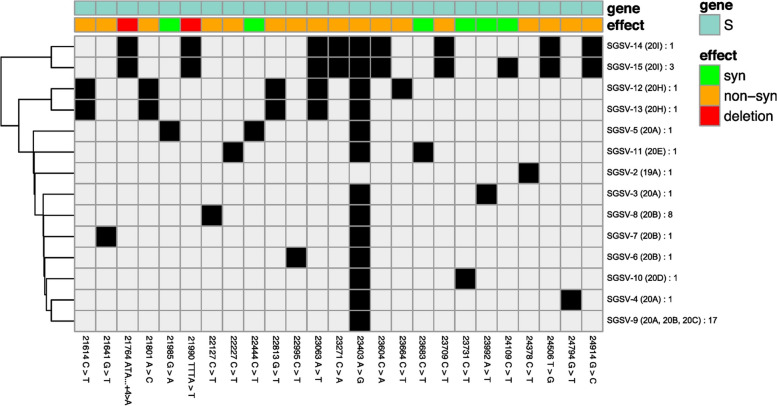


**Correct Figure 5**